# Severe hemoptysis in post-tuberculosis bronchiectasis precipitated by SARS-CoV-2 infection

**DOI:** 10.1186/s12890-020-01285-6

**Published:** 2020-09-14

**Authors:** Julien Lopinto, Marion Teulier, Audrey Milon, Guillaume Voiriot, Muriel Fartoukh

**Affiliations:** 1 Intensive Care Unit, Tenon Hospital, Assistance Publique – Hôpitaux de Paris, 4, rue de la Chine, 75020 Paris, France; 2 Department of Radiology, Tenon Hospital, Assistance Publique – Hôpitaux de Paris, Paris, France

**Keywords:** SARS-CoV-2, Severe hemoptysis, Bronchiectasis, Case report

## Abstract

**Background:**

Since the beginning of SARS-CoV-2 outbreak in China, severe acute respiratory syndrome has been widely descripted. Hemoptysis has rarely been observed in SARS-CoV-2 infection.

We report here a case of severe hemoptysis in post-tuberculosis bronchiectasis precipitated by SARS-CoV-2 infection and managed in a referral center.

**Case presentation:**

A 58-year-old man was admitted to our intensive care unit for severe hemoptysis with history of post-tuberculosis bronchiectasis. At ICU admission the patient had fever and severe acute respiratory failure requiring high flow oxygen therapy. Respiratory tract sampling was positive for SARS-CoV-2. Multi-detector computed tomography angiography pointed out localized bronchiectasis on the left lower lobe and enlarged left bronchial and phrenic arteries; bronchial arteriography with distal embolization was performed with favorable outcome and no bleeding recurrence.

**Conclusions:**

To our knowledge, this is the first case of acute exacerbation of bronchiectasis related to SARS-CoV-2 infection and complicated by severe hemoptysis. Whether the virus may play a role in the dysregulation of airway haemostasis, and contribute to episodes of hemoptysis in patients with chronic pulmonary diseases and predisposing factors might be investigated.

## Background

Severe hemoptysis remains a challenging condition for physicians because of the variety of etiologies and a large panel of clinical presentation. Acute lower respiratory tract infection is one of the conditions of severe hemoptysis in chronic pulmonary disease, acting like an acute bronchial inflammation trigger. Since the beginning of SARS-CoV-2 outbreak in China, severe acute respiratory syndrome has been widely descripted [1–3] and hemoptysis has rarely been observed in this condition [1, 2, 4, 5].

We report here a case of acute exacerbation of post-tuberculosis bronchiectasis precipitated by SARS-CoV-2 infection complicated by severe hemoptysis and managed in a referral center.

## Case presentation

**Fig. 1:**
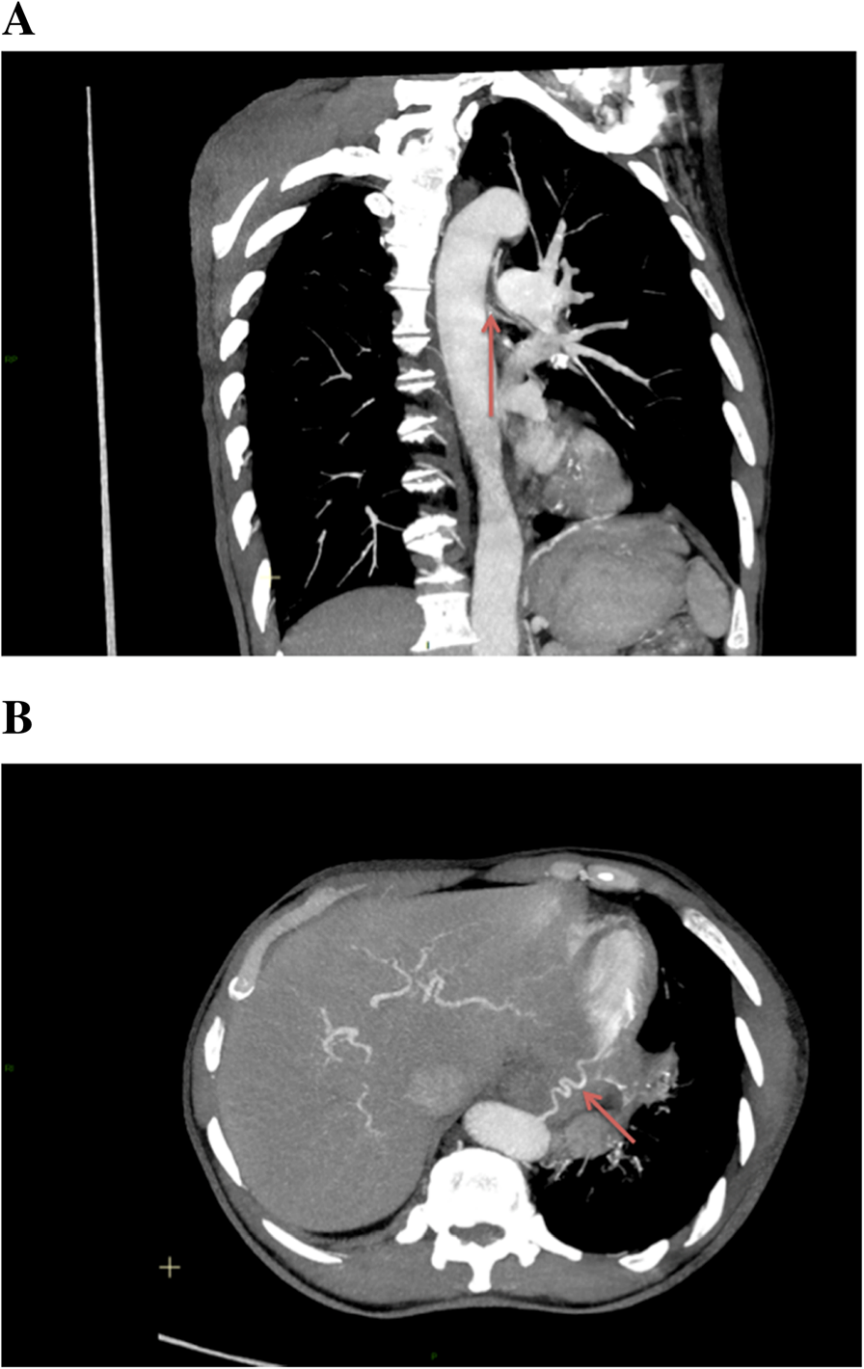
**a** Coronal CT section showing enlarged left bronchial artery (red arrow). **b** Axial CT section showing enlarged left phrenic artery (arrow)

A 58-year-old man was admitted to our intensive care unit (ICU) for severe hemoptysis. He had a history of post-tuberculosis bronchiectasis responsible for several episodes of hemoptysis of low abundance 3 years ago that resolved with medical measures. He was treated by rivaroxaban for a pulmonary embolism diagnosed within the two preceding months. Three days before ICU admission, he presented fever, cough, increased expectoration, shortness of breath, myalgia and asthenia. He had not travelled in Asia, but reported a contact in the preceding week with a colleague who had been tested positive for SARS-CoV-2. He presented acute massive hemoptysis (> 200 ml) with increased dyspnea and was referred to the Emergency Department of our hospital. A multi-detector computed tomography angiography pointed out poorly defined disseminated centrilobular nodules associated with localized bronchiectasis in the left lower lobe, enlarged left bronchial (Fig. 1a) and phrenic (Fig. 1b) arteries. On ICU admission, the patient had fever (39 °C) and severe acute respiratory failure requiring high flow oxygen therapy. Respiratory tract sampling was positive for SARS-CoV-2, without any bacterial or viral co-infection. Respiratory tract culture was negative for *Aspergillus sp* and *Mycobacterium tuberculosis*. Fungal sensitization was also considered but serum Aspergillus-specific IgE level was low.

Other relevant biochemical parameters were assessed: CRP was measured at 256 mg/ L, lactate dehydrogenase level was low 237 UI/ L and D-Dimere level was 1228 ng/ mL.

The anti Xa activity was measured at 20 ng/ml. A bronchial arteriography with distal embolization of both left bronchial and phrenic arteries was performed, using micro coils. Bleeding did not recur thereafter, and the outcome was favorable with no complications of the vascular interventional procedure, and hospital discharge at D14.

## Discussion and conclusions

Severe acute respiratory syndrome is known to be caused by SARS-CoV-2 since the beginning of the outbreak in December 2019 in China [1–3]. Hemoptysis has been rarely reported during SARS-CoV-2 infection [1, 2, 4, 5] (Table 1).

**Table 1:** Hemoptysis in SARS-CoV-2 infection in retrospective analysis in China

	Guan and al. NEJM 2020 [1]	Huang and al. LANCET 2020 [2]	Chen and al. BMJ 2020 [4]	Xu and al. BMJ 2020 [5]
All patients	1099	41	274	62
Hemoptysis, n (%)	10 (0.9)	2 (5)	7 (3)	2 (3)

Acute lower respiratory tract infection related to bacteria or virus is one of the conditions of severe hemoptysis, and may threaten life. In our case, the radiological pattern of SARS-CoV-2 pneumonia was consistent with a viral bronchial and pulmonary involvement, as already described, and slightly differed from that of the typical bilateral multi-lobar ground glass opacities with peripheral or posterior distribution (see Figure in Electronic Supplemental Material, ESM).

The patient had a history of post-tuberculosis bronchiectasis, pulmonary embolism and oral anticoagulation: these are all common risk factors for hemoptysis.

In our case the clinical presentation was consistent with influenza-like illness and the high abundance of bleeding differed from patient’s previous bronchiectasis exacerbations: all these observations might suggest here the role of SARS-CoV-2 as precipitating but not coincidental factor in this exacerbation.

In our opinion and in accordance with recent studies [1, 2, 4, 5] (Table 1), SARS-CoV-2 infection should not been considered as a common general risk factor of hemoptysis but could be a precipitating factor of chronic pulmonary disease exacerbation with hemoptysis.

One may hypothesize that the virus may have triggered acute bronchial inflammation and exacerbation of the chronic bronchial disease, ultimately leading to the rupture of the regional hyper vascularization of enlarged systemic arteries associated with bronchiectasis.

To our knowledge, this is the first case reported of acute exacerbation of post-tuberculosis bronchiectasis precipitated by SARS-CoV-2 infection and complicated by severe hemoptysis. Whether the virus may play a role in the dysregulation of airway haemostasis, and contribute to episodes of hemoptysis in patients with chronic pulmonary diseases and predisposing factors might be investigated.

## Supplementary information

**Additional file 1.** Figure Axial CT section showing localized bronchiectasis on the lower left lobe (blue arrow) and viral involvement with ground glass opacities (red arrow).

## Data Availability

All data are presented in the manuscript.

## Abbreviations

ICU: Intensive care unit
CRP: C-reactive protein
