# Supplementary material for: Severe hemoptysis in post-tuberculosis bronchiectasis precipitated by SARS-CoV-2 infection
Source: BMC Pulm Med. 2020 Sep 14;20:244. doi: 10.1186/s12890-020-01285-6 (PMC7488635; doi:10.1186/s12890-020-01285-6)

**Supplemental material**

**Figure**

Axial CT section showing localized bronchiectasis on the lower left lobe (blue arrow) and viral involvement with ground glass opacities (red arrow)


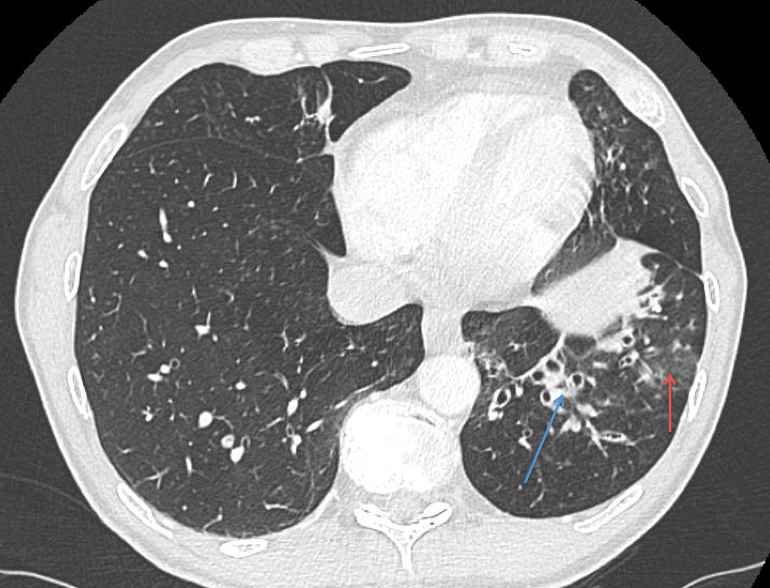

Supplement: Supplementary file 1 — Additional file 1. Figure Axial CT section showing localized bronchiectasis on the lower left lobe (blue arrow) and viral involvement with ground glass opacities (red arrow). [file 12890_2020_1285_MOESM1_ESM.docx]
